# Sensitivity of the EQ-5D-5L for fatigue and cognitive problems and their added value in Q-fever patients

**DOI:** 10.1007/s11136-021-03069-9

**Published:** 2022-01-29

**Authors:** A. J. L. M. Geraerds, Suzanne Polinder, Inge Spronk, Alfons G. M. Olde Loohuis, Annemieke de Groot, Madelon B. Bronner, Juanita A. Haagsma

**Affiliations:** 1 Department of Public Health, Erasmus MC, University Medical Center Rotterdam, P.O. Box 2040, 3000 CA Rotterdam, The Netherlands; 2 Association of Dutch Burn Centres, Maasstad Hospital, Rotterdam, The Netherlands; 3 Q-support, ʹs-Hertogenbosch, The Netherlands

**Keywords:** EQ-5D, Q-fever, Sensitivity, Fatigue, Cognition

## Abstract

**Purpose:**

Measuring health-related quality of life (HRQoL) with the EQ-5D-5L might lack sensitivity for disease-specific health complaints. This cross-sectional study analyzed whether fatigue and cognitive problems are captured by the EQ-5D-5L in a Q-fever patient population with persistent fatigue/cognitive problems, and whether addition of fatigue/cognition improved the explained variance for HRQoL.

**Methods:**

A Dutch sample of Q-fever patients filled out the EQ-5D-5L and EQ VAS, the fatigue subscale of the Checklist Individual Strength, and a cognition dimension in the EQ-5D-5L format. The extent to which fatigue and cognition were captured by the EQ-5D-5L was determined based on distributional effects, head-to-head comparisons, Spearman rank correlation coefficients, and regression analyses. Explanatory power was determined of the EQ-5D-5L for the EQ VAS with and without a fatigue and cognition dimension.

**Results:**

Out of 432 respondents, 373(86%) reported severe fatigue, 387(90%) cognitive problems. EQ-5D-5L utility and EQ VAS scores of respondents reporting severe fatigue/cognitive problems were significantly lower. Fatigue was strongly correlated with EQ-5D-5L dimensions usual activities and pain/discomfort (*r* = 0.602 and *r* = 0.510) and moderately with other EQ-5D-5L dimensions (*r* = 0.305–0.476). Cognition was strongly correlated with usual activities (*r* = 0.554) and moderately with other dimensions (*r* = 0.291–0.451). Adding fatigue to the EQ-5D-5L increased explanatory power for the EQ VAS with 6%.

**Conclusion:**

Fatigue and cognitive problems in Q-fever patients were partially captured by the EQ-5D-5L dimensions. The addition of fatigue to the EQ-5D-5L slightly improved explained variance for the EQ VAS. This potentially also accounts for patients who experience sequelae of other infectious diseases, such as COVID-19.

**Supplementary Information:**

The online version contains supplementary material available at 10.1007/s11136-021-03069-9.

## Background

Infectious diseases, such as Q-fever or COVID-19, are caused by organisms such as bacteria, viruses, fungi, or parasites, and can lead to illness, disability, and even death [1, 2]. In the Southeast region of the Netherlands, there has been an outbreak of Q-fever from 2007 until 2011 with over 3000 notified cases [3].

Approximately, 40% of all people that get infected with Q-fever experience health complaints that can vary in duration from days to lifelong [4]. One of the eminent characteristic health complaints of Q-fever is fatigue [5]. Ten years after infection, approximately 90% of Q-fever patients reported fatigue complaints [6], which is more than respondents who were not exposed to Q-fever [7].

Apart from fatigue, a vast majority of Q-fever patients also reported long-term problems with cognition [6, 8]. Two studies reported reduced work participation in Q-fever patients twelve months after infection, mainly due to concentration-, memory problems [9], and reduced cognitive functioning in Q-fever patients 5–9 years after infection, compared to the general population [10].

The magnitude of the current COVID-19 pandemic and the large percentage of COVID-19 patients experiencing long-term consequences of COVID-19, including fatigue and cognitive problems, underline the impact that long-term consequences of an infectious disease can have at population level [11, 12]. Insight into the magnitude of the impact of long-term sequelae of infectious disease can be assessed by investigating a patients’ health-related quality of life (HRQoL).

One of the most commonly used generic HRQoL measures is the EQ-5D [13]. The EQ-5D is a brief self-assessment measurement instrument that consists of five dimensions, with a response scale of five answer options in the 5L version [13, 14]. The EQ-5D-5L is widely used for, among other things, measuring utility for a cost-utility analysis, with the aim to inform policy-makers on allocation of healthcare resources. However, it has been argued that its brevity may limit the sensitivity to change and content validity in some (patient) populations, and therefore limit its’ ability to measure for example long-term consequences of infectious diseases [15, 16]. Moreover, the question rises whether certain aspects of health, such as fatigue and cognition, are sufficiently covered by the EQ-5D, or whether they should be added to the EQ-5D [17]. Especially in patients with infectious disease, who frequently experience persistent fatigue, cognitive problems, or both, it is crucial that the effects of these problems are captured by the HRQoL measurement instrument.

To date, there is only limited evidence available on the sensitivity of the EQ-5D for fatigue and cognitive problems in patients who experience these problems due to an infectious disease, such as Q-fever. Therefore, this study investigated whether fatigue and cognitive problems are captured by the EQ-5D-5L dimensions in a cohort of Q-fever patients. Furthermore, this study analyzed whether the addition of fatigue and cognition improved the extent to which the EQ-5D-5L captured HRQoL in Q-fever patients.

## Methods

### Study population

This study was conducted with self-reported cross-sectional data from Q-fever patients. An online survey was sent to Q-fever patients in December 2018 by a patient organization, Q-uestion, and a governmental support organization for Q-fever patients, Q-support [6]. Inclusion criteria were: a member of one of the patient organizations; adult (age ≥ 18 years old); ability to read Dutch. To receive support from Q-support, patients had to present a positive test result of a Q-fever infection. The study was approved by the Medical Ethics Review Board of the Erasmus Medical Center (MEC-2018-1605), and informed consent was retrieved from all respondents.

### Measures

The online survey included questions on socio-demographic characteristics, medical information, HRQoL, fatigue and cognition. Socio-demographic characteristics included age, sex and educational level (categorized in three categories: low, middle and high education) [18].

Patients were classified according to three diagnosis groups based on their self-reported diagnosis: chronic Q-fever (CQ), Q-fever fatigue syndrome (QFS), and patients who experience QFS-like disease (QLD). Furthermore, medical data included hospitalization (yes/no) during acute infection.

Patients were asked to report their HRQoL on the EQ-5D-5L and visual analogue scale (EQ VAS). The EQ-5D-5L consists of five dimensions: mobility, self-care, usual activities, pain/discomfort and anxiety/depression [14]. Each dimension is operationalized in one item with five response levels: no problems, slight problems, some problems, severe problems and extreme problems/unable to [13]. The EQ VAS consists of one question where respondents rate their health state on a scale from 0 (worst imaginable health state) to 100 (best imaginable health state), and is often used to determine to what extent the EQ-5D captures HRQoL [17].

The survey also informed on problems with fatigue, using the subscale ‘subjective experience of fatigue’, of the Checklist Individual Strength (CIS) [19]. The CIS fatigue scale is a validated instrument that consists of eight items that inform on different aspects of fatigue [19, 20]. Response options consist of a Likert scale from ‘Yes’ (score 1) to ‘No’ (score 7), with no description in words of the answer options in between score 1 and 7. Scores were recoded so that a score 7 always indicated fatigue. The CIS fatigue score was categorized in two groups: no to moderate fatigue (CIS fatigue score < 35), and severe fatigue (CIS fatigue score ≥ 35) [21]. Since some fatigue complaints are common in a general population, and therefore, not necessarily indicative of more problems than ‘normal’, no to moderate fatigue was considered one group [22].

In addition, a frequently used item on cognitive problems in EQ-5D-5L format was included in the survey, informing on problems with memory/understanding/coherence/thinking. The item has been tested in multiple studies on its psychometric performance [23, 24]. The wording of the item was: ‘Cognition, such as memory, understanding, concentration, thinking’. The answer options were the same as the answer options of the EQ-5D-5L items. Degree of cognitive problems was categorized in two groups: no cognitive problems (score 1), and cognitive problems (score 2–5). We will refer to this cognition item as the cognition dimension.

### Data analyses

Data analyses were performed using SPSS version 25 (Statistical Product and Service Solutions, Chicago, Illinois, USA). Respondents were included in the analyses if the EQ-5D, EQ VAS, CIS fatigue items and the cognition dimension had been completed.

Socio-demographic characteristics were presented for the whole population and for subgroups based on fatigue and cognitive problems. Distributional effect was determined by defining the proportion of perfect health profiles among all observed health profiles. A health profile was formed by combining the responses to the EQ-5D into a profile, e.g. ‘13252’. A perfect health profile consisted of ‘no problems’ on all EQ-5D dimensions (‘11111’), and a higher proportion of perfect health profiles indicated more ceiling effect. Utility scores were calculated using the Dutch value set for the EQ-5D-5L [25].

Mean and standard deviation from utility scores and EQ VAS were compared between groups based on the presence of severe fatigue, cognitive problems, or both.

A head-to-head comparison was performed between dimensions of the EQ-5D-5L and fatigue. The percentage of respondents with corresponding answers on fatigue and on EQ-5D dimensions was assessed. Corresponding answers referred to reporting no or mild problems on the fatigue scale in combination with reporting no problems on the EQ-5D dimension, or the opposite (severe fatigue in combination with reporting problems on the EQ-5D dimension). The same assessment was performed for the cognition dimension with each EQ-5D dimension. However, since the cognition dimension is designed in a similar way as the EQ-5D dimensions, corresponding answers were defined as reporting no problems on the cognition dimension in combination with no problems on the EQ-5D dimension, and reporting problems on the cognition dimension in combination with problems on the EQ-5D dimension. Furthermore, dominance of dimensions was analyzed by determining whether problems on the fatigue or cognition dimension were always associated with problems on another dimension.

Convergent validity between the EQ-5D dimensions with the CIS fatigue score and the cognition dimension was determined using Spearman rank correlation coefficient. A correlation of 0.1–0.29 was considered weak, 0.3–0.49 moderate, and ≥ 0.5 was considered strong [26]. For fatigue, a moderate to strong correlation was expected for all dimensions, because both physical and mental problems are likely to be worsened by fatigue [27]. For cognition, a strong correlation was expected with usual activities, as usual activities require concentration (for example for work), which is likely to be strongly related to cognitive problems [28]. In addition, a moderate to strong correlation was expected with pain/discomfort, as already identified in previous studies [29, 30], and with anxiety/depression, because especially mental problems are likely to be affected by cognitive problems. Moreover, anxiety/depression could lead to cognitive impairment [31].

Explanatory power of the EQ-5D for fatigue and cognition was determined using multiple linear regression analyses, as the assumptions of linear regression were met, to gain insight in the association, if any, between fatigue and cognition and the EQ-5D dimensions. Dummy variables were created for each response level for each EQ-5D dimension, except for ‘no problems’, which was used as reference category. Explained variance was reported for the full model, and unstandardized beta were reported for independent variables with a statistically significant effect (*p* < 0.05).

Furthermore, explanatory power of the EQ-5D (with fatigue/cognition) for EQ VAS was also determined using multiple linear regression analyses, to gain insight in the added value of fatigue and cognition to the measurement of HRQoL, measured with the EQ VAS. Dummy variables for each response level, except for ‘no problems’, were used as independent variables.

## Results

Overall, 478 out of 880 invited Q-fever patients responded to the survey (54.3%), of whom 432 patients filled out all relevant items and were therefore included in this study. Respondents were on average 56 years old, and approximately half of them were female (48%) (Table 1). More than half of the respondents reported a diagnosis of QFS (59%), and approximately one quarter of respondents reported hospitalization for Q-fever (23%).

**Table 1:** Characteristics of study population

	All responders	Subgroups based on fatigue		Subgroups based on cognitive problems	
		No/mild fatigue	Severe fatigue	No cognitive problems	Cognitive problems
*N*	432	59	373	45	387
Age mean(SD)	56.1 (13.3)	59.9 (13.4)	55.5 (13.2)	61.8 (11.7)	55.4 (13.3)
Female *N*(%)	208 (48%)	19 (32%)	189 (51%)	20 (44%)	188 (49%)
Education level *N*(%)					
Low	127 (29%)	14 (24%)	113 (30%)	14 (31%)	113 (29%)
Medium	158 (37%)	20 (34%)	138 (37%)	13 (29%)	145 (38%)
High	147 (34%)	25 (42%)	122 (33%)	18 (40%)	129 (33%)
Diagnosis *N*(%)					
Chronic Q-fever	46 (11%)	12 (20%)	34 (9%)	9 (20%)	37 (10%)
Q-fever fatigue syndrome (QFS)	255 (59%)	17 (29%)	238 (64%)	13 (29%)	242 (63%)
QFS-like disease	131 (30%)	30 (51%)	101 (27%)	23 (51%)	108 (28%)
Hospitalization *N*(%)	97 (23%)	17 (29%)	80 (21%)	13 (29%)	84 (22%)
Health profile ‘11111’ *N*(%)	20 (5%)	18 (31%)	2 (1%)	11 (24%)	9 (2%)
Utility score	0.53 (0.29)	0.81 (0.18)	0.49 (0.28)	0.79 (0.19)	0.50 (0.28)
EQ VAS	47 (19.8)	70 (15.3)	44 (18.0)	65 (16.9)	45 (19.1)

### Health outcomes

Respondents reported a mean EQ-5D-5L utility score of 0.53, and a mean EQ VAS of 47. Perfect health on the EQ-5D-5L was reported by 5% of respondents, indicating a small ceiling. Of the respondents, 86% reported severe fatigue, 90% cognitive problems (Table 1), and 80% reported both. Distribution of responses to the CIS fatigue items, on which severity of fatigue was based, can be found in Supplementary Information Fig. A1 and A2. Mean utility score was significantly higher (*p* < 0.001) for respondents with no or mild fatigue (EQ-5D-5L utility score = 0.81) compared to respondents with severe fatigue (EQ-5D-5L utility score = 0.49). A mean EQ-5D-5L utility score of 0.79 was found for respondents with no cognitive problems, versus a significantly lower (*p* < 0.001) EQ-5D-5L utility score of 0.50 for respondents with cognitive problems. Mean EQ VAS scores were in line with mean utility scores, with the highest mean EQ VAS for respondents with no or mild fatigue (EQ VAS = 70) and respondents with no cognitive problems (EQ VAS = 65), and the lowest VAS for respondents with severe fatigue (EQ VAS = 44) and respondents with cognitive problems (EQ VAS = 45).

### Distribution of responses

**Fig. 1:**
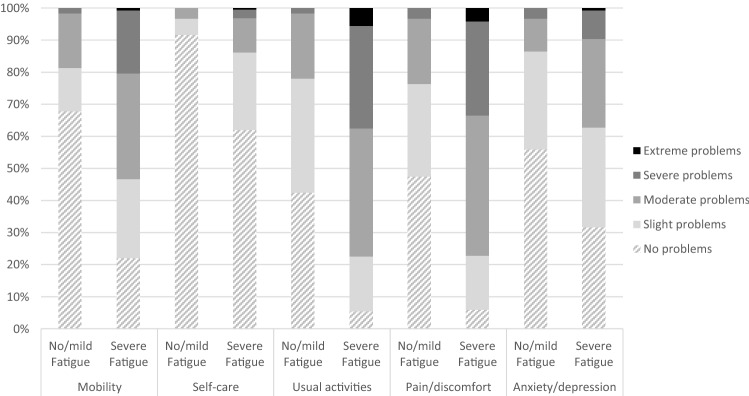
Distribution of responses on the EQ-5D-5L dimensions by severity level of fatigue (no/mild fatigue versus severe fatigue)

**Fig. 2:**
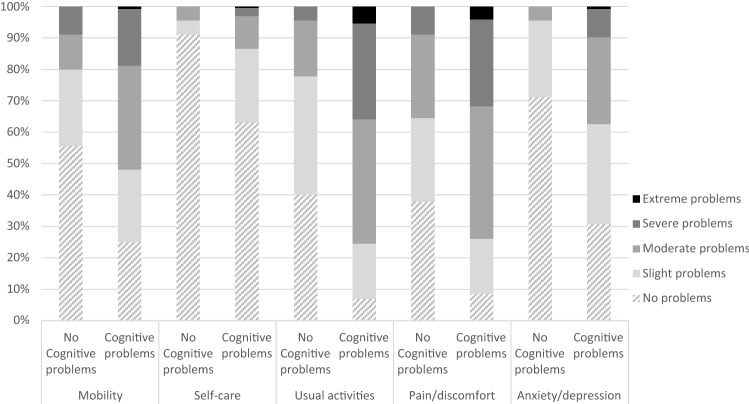
Distribution of responses on the EQ-5D-5L dimensions by severity level of cognitive problems (no cognitive problems versus cognitive problems)

Figures 1 and 2 present the distribution of responses for each EQ-5D-5L dimension for subgroups based on fatigue (1) and cognitive problems (2). The percentage of respondents reporting problems on a dimension is higher in the presence of fatigue or cognitive problems, for all dimensions. Most problems were reported on the usual activities and pain/discomfort dimension for both severe fatigue and cognitive problems. Least problems were reported with self-care for all respondents.

Reporting severe fatigue or cognitive problems was almost always (99% and 98% respectively) associated with problems on at least one EQ-5D dimension. However, reporting problems on at least one EQ-5D dimension was not always associated with severe fatigue (10% reported problems on the EQ-5D and no/mild fatigue). Similarly, for cognition it was found that 8% of respondents who reported problems on at least one EQ-5D dimension, reported no problems with cognition.

### Head-to-head comparison

Chi-square tests showed that fatigue and cognition were significantly related to all EQ-5D dimensions (*p* < 0.01), when testing on a dichotomized level. For all dimensions, except for self-care, > 50% of respondents reported corresponding answers for both fatigue and the cognition dimension with the EQ-5D (Table 2). The highest percentages of corresponding answers for fatigue and cognition with the EQ-5D dimensions were found for usual activities (fatigue: 88%; cognition: 88%) and pain/discomfort (fatigue: 88%; cognition 86%). The lowest percentages were found for self-care (fatigue: 45%; cognition: 43%). Furthermore, in 84% of the cases corresponding answers were found for fatigue and cognition.

**Table 2:** Head-to-head comparison of fatigue and the cognition dimension with the dimensions of the EQ-5D-5L

	Fatigue		Cognitive problems	
	No^a^ (%)	Yes^b^ (%)	No^c^ (%)	Yes^d^ (%)
*Mobility problems*				
No^c^	**40 (9)**	82 (19)	**25 (6)**	97 (23)
Yes^d^	19 (4)	**291 (67)**	20 (5)	**290 (67)**
*Self-care problems*				
No^c^	**54 (13)**	231 (54)	**41 (10)**	244 (57)
Yes^d^	5 (1)	**142 (33)**	4 (1)	**143 (33)**
*Usual activities problems*				
No^c^	**25 (6)**	20 (5)	**18 (4)**	27 (6)
Yes^d^	34 (8)	**353 (82)**	27 (6)	**360 (83)**
*Pain/discomfort problems*				
No^c^	**28 (7)**	22 (5)	**17 (4)**	33 (8)
Yes^d^	31 (7)	**351 (81)**	28 (7)	**354 (82)**
*Anxiety/depression problems*				
No^c^	**33 (8)**	118 (27)	**32 (7)**	119 (28)
Yes^d^	26 (6)	**255 (59)**	13 (3)	**268 (62)**
*Fatigue*				
No^a^	–	–	**18 (4)**	41 (9)
Yes^b^	–	–	27 (6)	**346 (80)**

Bold frequencies (percentages) represent corresponding answers

^a^No fatigue represents both no and mild fatigue (based on a CIS score < 35)

^b^Yes fatigue represents severe fatigue (based on a CIS score ≥ 35)

^c^No (problems) represents a score of 1 on the EQ-5D or cognition dimension

^d^Yes (problems) represents a score > 1 on the EQ-5D or cognition dimension

### Convergent validity

Strong correlation was found between fatigue and both the usual activities dimension (*r* = 0.602) and the pain/discomfort dimension (*r* = 0.510) (Table 3). Moderate correlation was found for fatigue with the remaining three dimensions: mobility, self-care, and anxiety/depression. Correlation of cognition with the EQ-5D dimensions was found to be strong for usual activities only (*r* = 0.554). Pain/discomfort and anxiety/depression showed a moderate correlation with cognition, and mobility and self-care a weak correlation. Fatigue and cognition were found to be moderately correlated (*r* = 0.476).

**Table 3:** Spearman’s rank correlation of EQ-5D-5L dimensions with fatigue and cognition

	Mobility	Self-care	Usual activities	Pain/discomfort	Anxiety/depression	Cognition
Fatigue	0.435**	0.382**	0.602**	0.510**	0.305**	0.476**
Cognition	0.291**	0.292**	0.554**	0.451**	0.374**	–

**Statistically significant at a 1% level (*p* < 0.01)

### Regression analyses

Multiple regression analyses showed that the EQ-5D-5L dimensions explained 40% of variance in fatigue, and approximately 34% of variance in cognition (Table 4). For fatigue, it was found that reporting complaints on any level of usual activities significantly increased complaints with fatigue, compared to no complaints on usual activities. Furthermore, the same effect was found for reporting moderate complaints with mobility (level 3), compared to no problems with mobility, and reporting moderate, severe or extreme pain/discomfort, compared to no pain/discomfort. For cognition, it was found that reporting moderate to extreme problems with usual activities was associated with reporting more severe cognitive problems, compared to no problems with usual activities. Similar results were found for anxiety/depression.

**Table 4:** Multivariable regression analyses of EQ-5D for fatigue and of EQ-5D for cognition

Fatigue^a^		Cognition^b^	
Independent variables	Unstandardized beta^c^ (95% CI)	Independent variables	Unstandardized beta^c^ (95% CI)
Constant	33.76 (31.87, 35.65)**	Constant	2.03 (1.83, 2.22)**
Mobility level 2	1.52 (− 0.80, 3.83)	Usual activities level 2	0.11 (− 0.20, 0.43)
Mobility level 3	2.50 (0.16, 4.84)*	Usual activities level 3	0.59 (0.29, 0.90)**
Mobility level 4	1.43 (− 1.50, 4.35)	Usual activities level 4	1.05 (0.70, 1.40)**
Mobility level 5	− 0.61 (− 12.89, 11.67)	Usual activities level 5	1.07 (0.56, 1.59)**
Usual activities level 2	3.99 (0.97, 7.01)*	Anxiety/depression level 2	0.03 (− 0.18, 0.24)
Usual activities level 3	8.55 (5.60, 11.50)**	Anxiety/depression level 3	0.35 (0.13, 0.57)**
Usual activities level 4	11.40 (8.05, 14.76)**	Anxiety/depression level 4	0.70 (0.38, 1.02)**
Usual activities level 5	12.92 (7.97, 17.86)**	Anxiety/depression level 5	1.39 (0.35, 2.42)**
Pain/discomfort level 2	1.51 (− 1.55, 4.57)		
Pain/discomfort level 3	3.36 (0.44, 6.29)*		
Pain/discomfort level 4	4.12 (0.74, 7.49)*		
Pain/discomfort level 5	5.56 (0.24, 10.87)*		
*Adjusted R*^*2*^	0.400	*Adjusted R*^*2*^	0.337
*F value*	15.38**	*F value*	11.94**

*CI* Confidence Interval, *MO* mobility, *SC* Self-care, *UA* Usual activities, *PD* Pain/Discomfort, *AD* Anxiety/Depression, *FA* CIS fatigue score, *CO* Cognition

*Statistically significant at a 5% level (*p* < 0.05)

**Statistically significant at a 1% level (*p* < 0.01)

^a^Range 8–56, higher score indicates more fatigue complaints

^b^Range 1–5, higher score indicates more cognitive problems

^c^Only statistically significant (*p* < 0.05) independent variables were presented

Comparing the explained variance of the EQ VAS by the EQ-5D-5L dimensions with and without fatigue and the cognition dimension, it was found that adding the fatigue score increased the explained variance with 6%, whereas adding the cognition dimension resulted in an increase of only 0.1% in explained variance (Table 5). Adding both fatigue and cognition to the EQ-5D resulted in a slightly lower percentage of increased explained variance than adding fatigue only (51.7% vs 51.5%).

**Table 5:** Explanatory power of EQ-5D with and without fatigue and cognition for EQ VAS

Dependent variable	Independent variables	Adjusted R^2^	F value
EQ VAS	MO-SC-UA**-PD*-AD**^a^	0.456	19.06**
	MO-SC-UA**-PD*-AD**-FA**^b^	0.517	23.00**
	MO-SC-UA**-PD*-AD**-CO^c^	0.457	16.14**
	MO-SC-UA**-PD*-AD**-FA**-CO^d^	0.515	19.31**

*MO* mobility, *SC* Self-care, *UA* Usual activities, *PD* Pain/Discomfort, *AD* Anxiety/Depression, *FA* raw CIS fatigue score (continuous), *CO* Cognition

*Statistically significant at a 5% level (*p* < 0.05)

**Statistically significant at a 1% level (*p* < 0.01)

^a^Levels that were significant: UA levels 3**, 4**, 5**; PD levels 4*, 5**; AD levels 3**, 4**

^b^Levels that were significant: UA levels 4**, 5**; PD level 5*; AD levels 3**, 4**

^c^Levels that were significant: UA levels 3**, 4**, 5**; PD levels 4*, 5**; AD levels 3**, 4**

^d^Levels that were significant: UA levels 4**, 5**; PD level 5*; AD levels 3**, 4**

## Discussion

### Main findings

This study analyzed the sensitivity of the EQ-5D-5L for fatigue and cognitive problems in a sample of Q-fever patients. The majority of respondents reported both severe fatigue and cognitive problems. Fatigue and cognitive problems were partially captured by the EQ-5D-5L dimensions, although fatigue was captured to a slightly larger extent than cognitive problems. Fatigue was strongly correlated with both usual activities and pain/discomfort, and moderately correlated with the remaining dimensions, as expected. Cognition was strongly correlated with usual activities only, and moderately with pain/discomfort and anxiety/depression, which was also expected. Explained variance in fatigue and cognition by the EQ-5D-5L was found to be slightly smaller for cognition than for fatigue (34% vs. 40%). Interestingly, only mobility level 3 had a significant effect on fatigue, whereas the effect of level 4–5 was not significant. Possibly, people who report severe problems/unable to walk refrain from moving around and therefore experience less fatigue, whereas people with moderate problems with walking might experience more fatigue as they keep walking and use more energy in walking due to their impairment. The explorative analyses on extension of the EQ-5D-5L with fatigue/cognition showed that the addition of fatigue increased the explained variance of the EQ-5D-5L for the EQ VAS, whereas adding the cognition dimension had almost no impact on the explained variance. This means that while cognitive problems are only partially captured by the EQ-5D-5L, the five dimensions of the EQ-5D-5L represent cognition with respect to HRQoL, when measured with the EQ VAS. Fatigue, on the other hand, is less well represented in the EQ-5D-5L dimensions, since inclusion of fatigue improved the explained variance of the EQ-5D-5L for HRQoL. Furthermore, addition of both fatigue and cognition to the EQ-5D in the regression analyses resulted in a slightly lower explained variance than adding fatigue only, potentially due to the high correlation between fatigue and cognition.

### Comparison to previous studies

Fatigue, both in positive terminology (energy) and negative terminology (fatigue/tiredness) has been found to be missing in the EQ-5D in multiple studies [32, 33]. When included, many respondents reported problems on this dimension [34, 35]. Considering the extent to which fatigue is captured by the EQ-5D, a study by Spronk et al. reported small to moderate correlation of fatigue with the EQ-5D-5L in a general population, using the fatigue item of the Rivermead Post-Concussion Symptoms Questionnaire (RPQ) [36]. Respondents with at least one chronic condition had a stronger correlation between fatigue and the EQ-5D dimensions than respondents with no chronic disease, but the reported correlation was not as strong as the correlations for our Q-fever population [36]. This indicates that fatigue is better captured by the EQ-5D dimensions in a population with more fatigue complaints. However, it should be taken into account that the study by Spronk et al. used a different item to measure fatigue, which potentially measured different aspects of fatigue. Considering the explanatory power of the EQ-5D, another study reported that although respondents frequently reported problems on energy, it did not add to the explanatory power of the EQ-5D-3L, probably due to high correlation with emotional functions [16]. This was not in line with our findings, as we identified an increase in explanatory power of the EQ-5D-5L for the EQ VAS when including fatigue. However, our study added only fatigue as independent variable, whereas the study by Jelsma et al. [16] added more dimensions, which might have been overlapping with the energy dimension. Moreover, an energy dimension may not cover the same concept as fatigue. In addition, our study used the 5 level version of the EQ-5D. Comparing this finding to our regression analyses for the EQ VAS with both fatigue and cognition added to the EQ-5D, it is in line with the findings of Jelsma et al. as no additional explanatory power was found when adding both the cognition and fatigue dimension compared to only adding fatigue. This may have been caused by an interaction between fatigue and cognition, which was also in line with the findings of Jelsma et al. [16]. Moreover, a moderate correlation was found between fatigue and cognition, which supports the suspicion of a potential interaction. In addition, fatigue was based on eight items in our study, which were framed differently than the EQ-5D dimensions, instead of one bolt-on dimension in EQ-5D format. This could potentially affect the sensitivity of the CIS fatigue score, as eight items are likely to be able to measure more aspects of fatigue than one bolt-on dimension in EQ-5D format. In addition, the CIS fatigue scale informs on fatigue complaints in the past two weeks, whereas a dimension in the EQ-5D format informs on health today. Therefore, a fatigue bolt-on might be less sensitive to change in fatigue complaints than de CIS fatigue score.

The addition of a cognition dimension to the EQ-5D has been studied more extensively than fatigue, and cognition has in many studies already been applied as an additional dimension of the EQ-5D [17]. However, while often suggested [16, 32, 33] and also tested for added value [23, 24, 28, 34, 37–40], the cognition dimension is not an official EQ-5D dimension. Therefore, it is relevant to analyze the extent to which cognition is captured in a patient population with cognitive problems. Previous studies reported little added value for the cognition dimension in a variety of patient- and general populations when added to the EQ-5D-3L [28, 37] and the EQ-5D-5L [23, 24]. Although the improvement in explanatory power of the EQ-5D with cognition dimension for the EQ VAS was negligible, our results showed that cognition was captured partially in the EQ-5D dimension. This was in line with findings of the previously mentioned studies. The addition of the cognition item did not improve the explained variance of the EQ-5D-5L for the EQ VAS, although our findings indicated that cognition is not fully captured in the EQ-5D dimensions. This can be explained by the fact that the EQ VAS was used to represent HRQoL, due to the absence of a golden standard for HRQoL. It should be noted, however, that the EQ VAS has been argued to represent a broader underlying construct of health [41].

### Strengths and limitations

This study had some strengths and limitations. A major strength was that our study sample consisted of Q-fever patients who frequently reported fatigue and cognitive problems. This allowed us to investigate the sensitivity of the EQ-5D-5L for these key aspects of infectious disease sequelae.

A limitation of our study is that it is unknown whether respondents filled out the questionnaires by themselves. Approximately a quarter of respondents reported severe cognitive problems. Cognitive problems are likely to have affected the ability to fill out the questionnaire, which consisted of more than fifty questions. Therefore, responses might actually represent proxy responses. Proxy responses are responses about a patient, given by someone else (e.g. a family member). Previous studies showed that proxy responses can differ randomly from patient responses, and should therefore be interpreted with caution [42, 43].

Furthermore, it should be noted that data collection may have suffered from selection and non-response bias. Surveys were send to Q-fever patients by two Q-fever patient organizations, which might have led to a selection bias. In the Netherlands, there have been approximately 4300 notified cases of Q-fever between 2007 and 2018, whereas 880 (20%) was invited for our survey through the patient and governmental organizations. Possibly, only Q-fever patients with more severe complaints were members of one of the patient organizations. This is illustrated by the large percentage of patients with QFS (59%), whereas a systematic review indicated that approximately 20–30% suffers from QFS [44]. However, the governmental patient organization targets patients with long-term complaints. Since approximately 20–30% suffers from QFS, which leads to long-term complaints, the 20% of all notified cases that was reached should comprise a large proportion of all Q-fever patients with long-term complaints. Furthermore, potentially only Q-fever patients with ongoing complaints filled out the survey. On the other hand, patients with severe fatigue and cognitive problems might have been unable to fill out the questionnaire. There is no certainty about the direction of the potential selection and non-response bias. However, we believe that the outcomes of our analyses were not affected by this limitation, as representativeness of the study population was not required for our research question.

### Implications for future research

The results of our study suggested that fatigue was moderately captured in the EQ-5D-5L, but inclusion of a fatigue dimension in the EQ-5D could potentially improve the coverage of HRQoL. More research on the addition of a fatigue item, preferably in the EQ-5D phrasing and format, could provide more certainty on whether or not an additional fatigue item is necessary to measure HRQoL in certain patient groups. Furthermore, it would be recommended to study the differences in wording and concept of a fatigue item, and the effect of the wording on the additional value of a fatigue item for the EQ-5D-5L. For example, does the wording or concept, such as fatigue, tiredness, energy or vitality affect the added value of a bolt-on dimension? In addition, a similar study could be performed in a population of COVID-19 patients, to test whether results hold for patients experiencing persistent sequelae of other infectious disease as well. Furthermore, the added value of an additional dimension to the EQ-5D-5L could also be tested using other data, such as the results of a time trade-off analyses, since the EQ VAS is no golden standard.

## Conclusion

In conclusion, fatigue and cognitive problems are partially captured by the EQ-5D-5L dimensions in a population of Q-fever patients who reported at a large-scale severe fatigue and cognitive problems. Adding a fatigue dimension to the EQ-5D-5L slightly increased the explained variance of the EQ VAS. In contrast to fatigue, adding a cognition dimension to the EQ-5D-5L had no effect on the explained variance. Therefore, addition of a fatigue dimension to the EQ-5D-5L might further increase the explained variance of the EQ-5D-5L for the EQ VAS in patients experiencing persistent sequelae of other infectious disease.

## Supplementary Information

Below is the link to the electronic supplementary material.

Supplementary file1 (PDF 614 KB)
