# Supplementary material for: Sensitivity of the EQ-5D-5L for fatigue and cognitive problems and their added value in Q-fever patients
Source: Qual Life Res. 2022 Jan 29;31(7):2083–92. doi: 10.1007/s11136-021-03069-9 (PMC8799410; doi:10.1007/s11136-021-03069-9)
Supplement: Supplementary file 1 — Supplementary file1 (PDF 614 KB) [file 11136_2021_3069_MOESM1_ESM.pdf]

Supplementary Information

Sensitivity of the EQ-5D-5L for fatigue and cognitive problems and their added value in Q-fever patients

Quality of Life Research

A.J.L.M. Geraerds, Suzanne Polinder, Inge Spronk, Alfons G.M. Olde Loohuis, Annemieke de Groot, Madelon B. Bronner, Juanita A. Haagsma

Erasmus MC, University Medical Center Rotterdam, Department of Public Health, The Netherlands.

a.geraerds@erasmusmc.nl

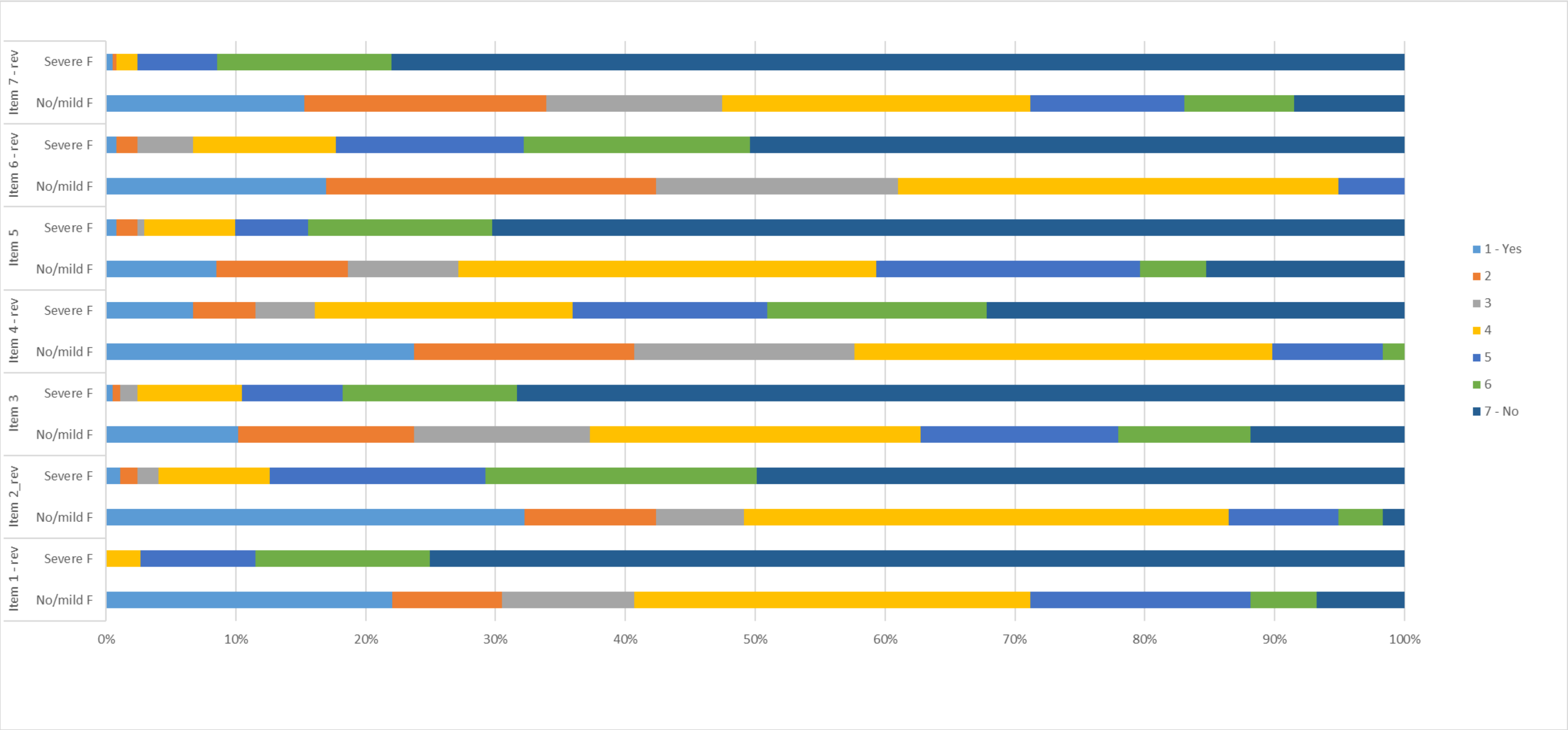

Figure A1: Distribution of responses on the CIS fatigue items per fatigue item for patients reporting no/mild fatigue and severe fatigue

NOTE1: Items with 'rev' were presented with reversed scoring, meaning that for all items a score of 7 represents fatigue, and a score of 1 represents no fatigue

NOTE2: Item 1: I feel tired; Item 2: Physically I feel exhausted; Item 3: I feel fit; Item 4: I feel weak; Item 5: I feel rested; Item 6: Physically I feel I am in a bad condition; Item 7: I get tired very quickly; Item 8: Physically I feel in a good shape

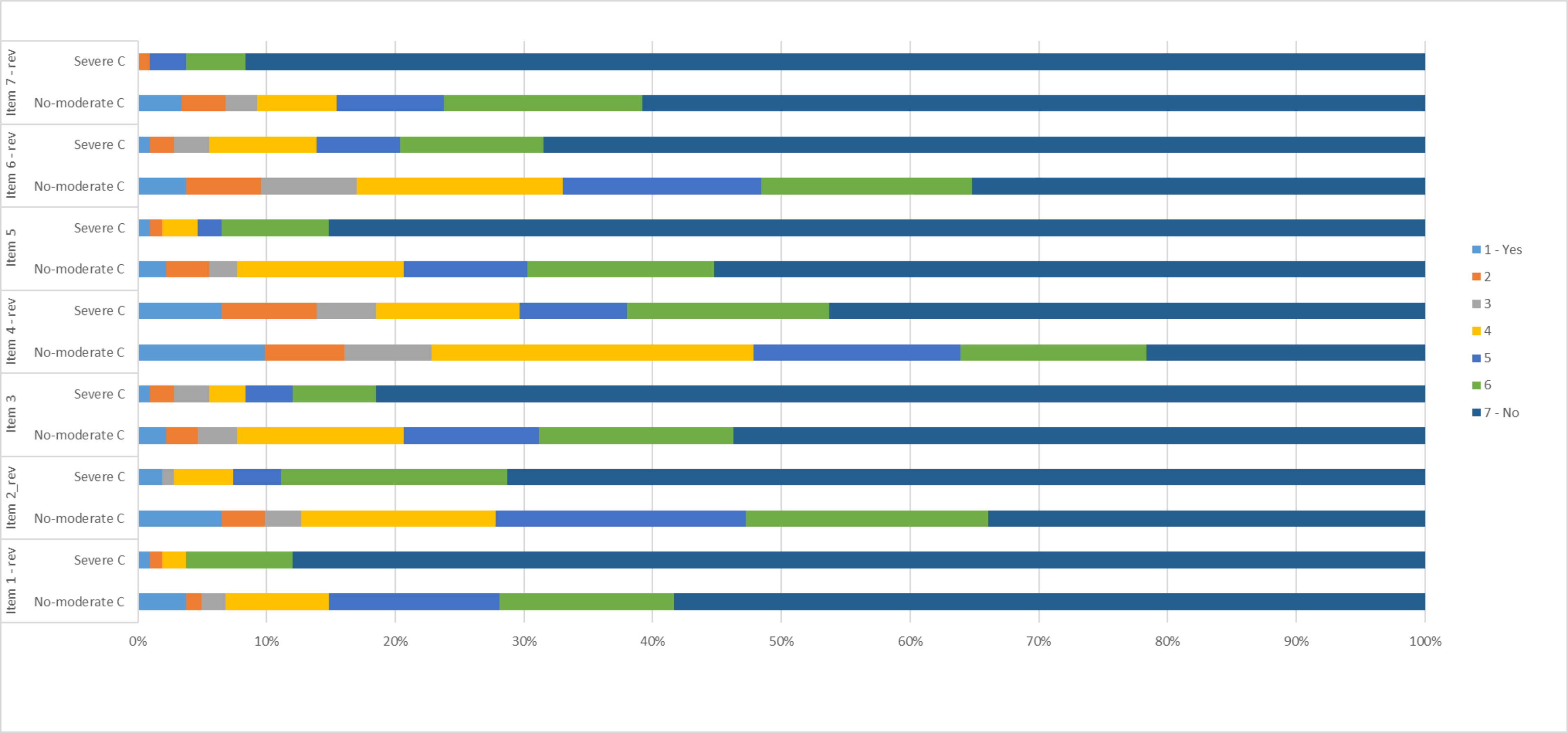

Figure A2: Distribution of responses on the CIS fatigue items per fatigue item for patients reporting no-moderate cognitive problems and severe cognitive problems

NOTE: Items with ‘rev’ were presented with reversed scoring, meaning that for all items a score of 7 represents fatigue, and a score of 1 represents no fatigue
